# Function and Characterization of an Alanine Dehydrogenase Homolog From *Nocardia seriolae*

**DOI:** 10.3389/fvets.2021.801990

**Published:** 2022-01-12

**Authors:** Guoquan Chen, Ziyang Tan, Yansheng Liu, Tingting Weng, Liqun Xia, Yishan Lu

**Affiliations:** ^1^Guangdong Provincial Key Laboratory of Pathogenic Biology and Epidemiology for Aquatic Economic Animals, Fisheries College of Guangdong Ocean University, Zhanjiang, China; ^2^Guangdong Provincial Engineering Research Center for Aquatic Animal Health Assessment, Shenzhen Public Service Platform for Evaluation of Marine Economic Animal Seedings, Shenzhen Institute of Guangdong Ocean University, Shenzhen, China

**Keywords:** *Nocardia seriolae*, alanine dehydrogenase, secreted protein, mitochondrial localization, apoptosis

## Abstract

Fish nocardiosis is a chronic, systemic, granulomatous disease in aquaculture. *Nocardia seriolae* has been reported to be one of the main pathogenic bacteria of fish nocardiosis. There are few studies on the associated virulence factors and pathogenesis of *N. seriolae*. Alanine dehydrogenase (ALD), which may be a secreted protein, was discovered by analysis using bioinformatics methods throughout the whole genomic sequence of *N. seriolae*. Nevertheless, the roles of ALD and its homologs in the pathogenesis of *N. seriolae* are not demonstrated. In this study, the function of *N. seriolae* ALD (NsALD) was preliminarily investigated by gene cloning, host cell subcellular localization, secreted protein identification, and cell apoptosis detection. Identification of the extracellular products of *N. seriolae via* mass spectrometry (MS) analysis revealed that NsALD is a secreted protein. In addition, subcellular localization of NsALD-GFP recombinant protein in fathead minnow (FHM) cells showed that the strong green fluorescence co-localized with the mitochondria. Moreover, apoptosis assays demonstrated that the overexpression of NsALD induces apoptosis in FHM cells. This study may lay the foundation for further exploration of the function of NsALD and facilitate further understanding of the pathogenic mechanism and the associated virulence factors of *N. seriolae*.

## Introduction

Fish nocardiosis, a chronic systemic granulomatous disease, has great influence on both marine and freshwater aquaculture industry (1, 2). *Nocardia salmonicida, Nocardia asteroids*, and *Nocardia seriolae* have been isolated from diseased fish and confirmed as the pathogenic bacteria of fish nocardiosis (3). Remarkably, *N. seriolae* has been most frequently reported as the main pathogen in the last 30 years. *Nocardia seriolae* can infect the immunodeficient fish *via* wounds, the gills, and feeds (4). The symptoms of diseased fish include skin ulceration and serious sarcoidosis caused by a mass of white nodules in the gills, spleen, liver, head kidney, and trunk kidney (5). According to reports, *N. seriolae* was able to infect about 42 kinds of marine and freshwater fish, such as blotched snakehead (*Channa maculata*), amberjack (*Seriola dumerili*), yellowtail (*Seriola quinqueradiata*), snubnose pompano (*Trachinotus blochii*), golden pompano (*Trachinotus ovatus*), largemouth bass (*Micropterus salmoides*), large yellow croaker (*Larimichthys crocea*), and red drum (*Sciaenops ocellatus*) (3, 6–8).

The virulence factors and pathogenic mechanisms of *N. seriolae*–host interaction are not fully studied. It was reported that the virulence of *Nocardia* species is related to their resistivity to oxidative damage of macrophages, inhibition of a combination of phagosomes and lysosomes, alteration of lysosomal enzymes in phagocytes, and neutralization of the acidification of the phagosome, and some extracellular products seemed to participate in the above processes in *Nocardia asteroide*s (9–12). Our previous studies also indicated that the MTSP3141, GluNS, NsHLP, PLC, SOD, robl/LC7, PTP, and NlpC/P60 of *N. seriolae* are able to lead to apoptosis in fish cells and are the potential virulence factors of *N. seriolae*. MTSP3141 and GluNS were also confirmed to be secreted proteins and mitochondrial targeting secretory proteins (MTSPs) (7, 13–15). The disease mechanisms of *Nocardia* sp. have been demonstrated to be varied and complicated and needed further clarification.

In this study, an alanine dehydrogenase (*ALD*) homolog of *N. seriolae* (*NsALD*) was amplified by gene cloning. Then, the secreted proteins were identified and the subcellular localization of NsALD and its involvement in disease mechanisms were evaluated. This study will put forward further clarification of the role of NsALD during infection and facilitate new insights into the molecular pathogenicity of *N. seriolae*.

## Materials and Methods

### Bacterial Strains, Cell Line, and Plasmids

*Nocardia seriolae* ZJ0503 strain (16), fathead minnow (FHM) cells (17), plasmid PEGFP-N1, plasmid PCDNA3.1-His A, and *Escherichia coli* DH5α were conserved in our laboratory. *Nocardia seriolae* ZJ0503 isolated from diseased fish was cultured in optimal medium to clone the *ALD* gene. *Escherichia coli* DH5α transformed by plasmid DNA was cultured to extract large numbers of endotoxin-free plasmids. FHM cells (17) transformed by endotoxin-free plasmids were cultured in Leibovitz's L15 medium with 10% fetal bovine serum for apoptosis assays. The endotoxin-free plasmids pEGFP-N1 and pCDNA3.1-His A were used for subcellular localization and overexpression, respectively.

### Gene Cloning and Sequence Bioinformatics Analysis of *NsALD*

*Nocardia seriolae* ZJ0503 was collected after 5 days of cultivation in optimal medium to extract genomic DNA using TIANamp Bacteria DNA Kit (Tiangen, Beijing, China). *NsALD* was cloned *via* PCR with two pairs of primers designed using Primer Premier 5.0 software, pEGFP**-**ALD-F/R and pcDNA**-**ALD-F/R, shown in Table 1. Based on the whole-genome sequence data of the *N. seriolae* strain ZJ0503 (accession no. NZ_JNCT01000022), BLAST sequence analysis was carried out through NCBI (http://www.ncbi.nlm.nih.gov/BLAST/). The amino acid sequence for NsALD was predicted and the physicochemical property predicted using ExPASy software (http://www.expasy.org/). Multiple sequence alignment was done with DNAMAN software. A biological evolutionary tree was constructed using the evolutionary analysis software MEGA 6.0. LocTree 3 (https://rostlab.org/services/loctree3/) and PSORT II Prediction (https://psort.hgc.jp/form2.html) were utilized to predict the subcellular localization and signal peptides.

**Table 1:** Primers used for gene cloning in this study.

**Primer name**	**Sequence (5^**′**^-3^**′**^)**	**Restriction enzyme**
pEGFP-ALD-F	GGAATTCATGCTGTTCGATAGCGGCATC	EcoR I
pEGFP-ALD-R	CCGACGTCGACTGGGAGGCGATGCGGGTCAC	Sal I
pcDNA-ALD-F	GGAATTCATGCTGTTCGATAGCGGCATC	EcoR I
pcDNA-ALD-R	CCGCTCGAGGGAGGCGATGCGGGTCACC	Xho I

### Plasmid Construction

Using the two pairs of primers listed in Table 1, the gene *NsALD* was cloned into pcDNA3.1/His A and pEGFP-N1 vectors to explore the molecular function and subcellular localization of NsALD *in vitro*. The constructed recombinant plasmids were subsequently confirmed by sequencing.

PCR was performed with TaKaRa Ex Taq^®^ polymerase using the following PCR program: pre-denaturation at 95°C for 5 min, 34 cycles at 95°C for 30 s, 55°C for 30 s, 72°C for 1 min, and a final extension at 72°C for 3 min. The PCR products of *NsALD* were electrophoresed on 1% agarose gel and purified using EasyPure PCR Purification Kit (TransGen, Beijing, China). The purified PCR products were digested by the corresponding restriction enzymes, ligated into the eukaryotic vectors pEGFP-N1 and pcDNA3.1/His A, and then transformed into competent *E. coli* DH5α cells. The different constructs were confirmed by corresponding restriction enzyme digestion (Table 1) and DNA sequencing by Guangzhou Sangon Biological Engineering & Technology and Service Co. Ltd. Finally, the recombinant plasmids named as pEGFP-ALD and pcDNA-ALD.

### Preparation and Identification of Extracellular Products

After culturing in optimal medium for 3–5 days, single colonies were selected to prepare bacterial suspension. Sterilized cellophane was laid flat on the modified medium plate as close to the medium as possible. Of the bacterial suspension, 100 μl was spread evenly on the medium covered with cellophane and incubated at 28°C for 3–5 days. The cellophane with colonies from the medium was removed with tweezers and the colonies on the cellophane rinsed into a sterile beaker with sterile phosphate-buffered saline (PBS). The collected liquid was transferred into a 50-ml centrifuge tube at 8,000 × *g* at 4°C for 20 min. After centrifugation, the supernatant was sterilized using a 0.22-μm microporous membrane and then transferred into a dialysis bag. Dialysis was performed at 4°C for 10–16 h in ultrapure water, during which the dialysate was changed 3 times. After dialysis, the samples were transferred into a 50-ml centrifuge tube and the liquid was frozen into solid at −80°C before freeze drying in a vacuum freeze dryer. The freeze-dried extracellular product samples were sent to a related biological company for protein shotgun LC-MS identification.

### Cell Transfection and Subcellular Localization

Plasmids pEGFP-ALD and pEGFP-N1 were extracted in large quantities *via* Endo-Free Plasmid Mini Kit I D6948 (Omega Bio-Tek, Norcross, GA, USA). Cell transfection was carried out to explore the subcellular localization of *NsALD* in host cells using the Lipofectamine 3000 reagent. At 48 h post-transfection (hpt), pEGFP-ALD-transfected FHM cells and pEGFP-N1-transfected cells were both stained with 4′,6-diamidino-2-phenylindole (DAPI) and Mito Tracker Red according to the protocols. After staining and washing with sterilized PBS, the cells were examined under a fluorescence microscope.

### Detection of Cell Apoptosis Induced by Overexpression of *NsALD* Protein

To determine whether the overexpression of NsALD induces apoptosis in fish cells, transient transfection of FHM cells was carried out using pcDNA-ALD as an experimental group or plasmid pcDNA3.1/His A as a control group. The overexpression of *NsALD* in FHM cells was verified by Western blot. Then, FHM cells were dyed with DAPI at 48 hpt and observed using a fluorescence microscope. Furthermore, the caspase-3 activity and mitochondrial membrane potential (ΔΨ_m_) of the transfected cells were detected, respectively (18) with a caspase-3 colorimetric assay kit K106-25 (BioVision, Milpitas, CA, USA) and a mitochondrial membrane potential assay kit with JC-1 (Beyotime, Shanghai, China).

### Quantitative Analysis of the mRNA Expression of Apoptosis-Related Genes

Quantitative PCR primers (Table 2) for the genes *Bcl-2, Bax, Bid*, and *Bad* of FHM cells were designed using Primer Premier 5.0 software. FHM cells transfected with pcDNA-ALD and pcDNA3.1/His A were collected at 0, 24, 48, and 72 hpt. Total RNA was extracted using the TransZol Up Plus RNA Kit (TransGen) and cDNA was synthesized with the EasyScript^®^ One-Step gDNA Removal and cDNA Synthesis SuperMix (TransGen) for real-time quantitative PCR (RT-qPCR) with PerfectStart^®^ Green qPCR SuperMix (TransGen). The effect of transfection on the expressions of apoptosis-related genes was calculated by LightCycler 96 software for RT-qPCR. Each test was performed with the β*-actin* gene as the internal control, and there were 3 replicates. The PCR reaction volume was 10 μl, 1 μl for each primer (10 μM), 1 μl for cDNA, 2 μl for PCR grade water, and 5 μl for Green qPCR SuperMix. The PCR procedures for the four apoptosis-related genes and β*-actin* were as follows: 95°C for 2 min, 40 cycles of 95°C for 75 s, and 55°C for 1 min.

**Table 2:** Primers used for qRT-PCR in this study.

**Primer name**	**Sequence (5^**′**^-3^**′**^)**	**Gene name**
Bad-F	TGATCCTTTCAGGCGGAGATCTCGC	Bad
Bad-R	CAGACTCTTTGTGACTCCAAAGGAA	
Bid-F	CTGCTTCTCCTTTCCTTCTTTGAGC	Bid
Bid-R	GATCAACTCAGCAGCCATATCCCTT	
Bax-F	TGGCACTGTTTCACCTCG	Bax
Bax-R	ATCCTCCTTGCTGTCTGATC	
Bcl-2-F	TGGGACTGTTTGCCTTCG	Bcl-2
Bcl-2-R	TCTGCCGCTGCATCTTTT	
β-actin-F	ACAATCAATACGGCTGCCATGG	β-actin
β-actin-R	TTGGCATACAGGTCCTTACTTACGT	

### Data Analysis

The comparative 2^(−ΔΔCt)^ method was used to calculate the relative expression levels of the four apoptosis-related genes. The data obtained were analyzed with one-way ANOVA using GraphPad Prism 8.0 software. The data were edited and plotted into histograms, and the expression of each apoptosis-related gene at 0 hpt was calculated as 1. Asterisks show statistical significance, as follows: *p* > 0.05, not significant; ^*^p < 0.05, significant; and ^**^p < 0.01, extremely significant.

## Results

### Sequence Characterization and Bioinformatics Analysis of *NsALD*

**Figure 1:**
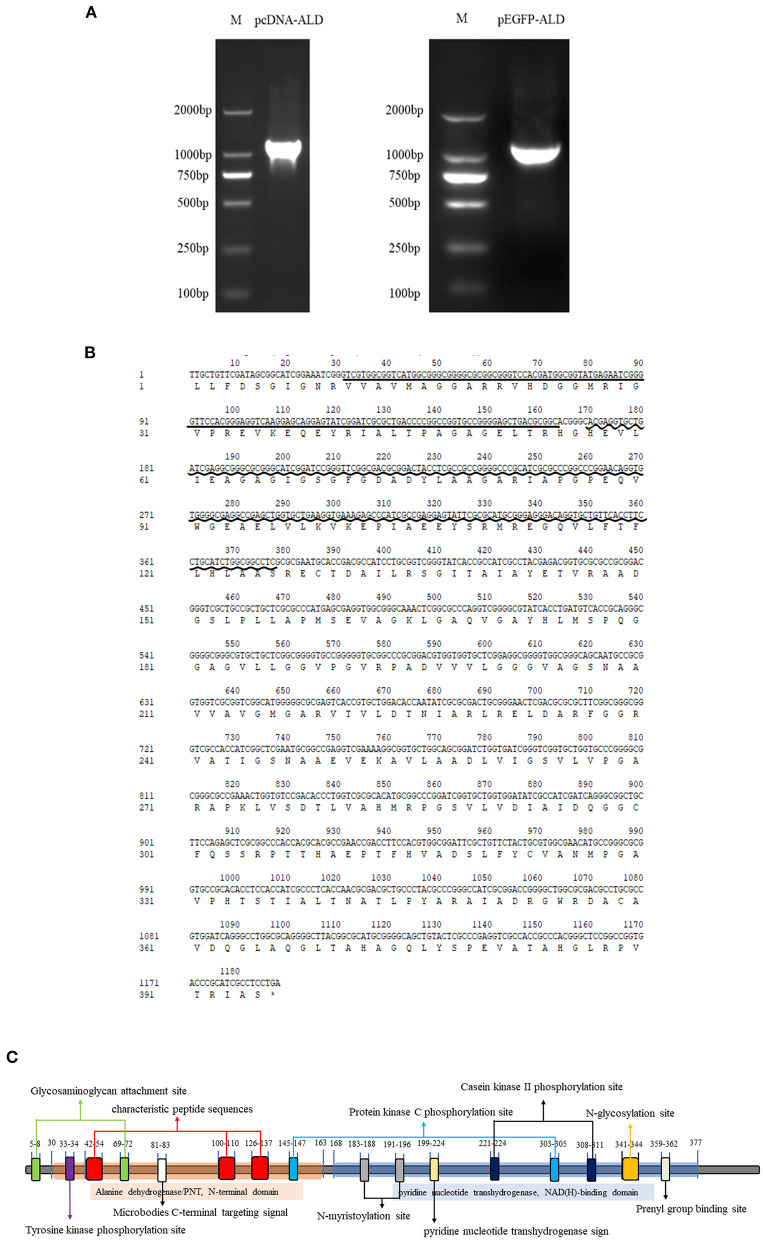
Sequence and structure analysis of the alanine dehydrogenase of *Nocardia seriolae* (NsALD). **(A)** NsALD was amplified *via* PCR using the primers pEGFP-ALD-F/R and pcDNA-ALD-F/R. **(B)** NsALD nucleic acid sequence and its derived amino acid sequence. The *straight line* indicates the ALD/PNT, N-terminal domain and the *wavy line* shows the ALD NAD-binding and catalytic domains. **(C)** Schematic representation of the prosites of protein NsALD. The NsALD protein comprised two complexity domains (30–163 and 168–377) and nine kinds of functional sites.

*NsALD* was cloned *via* PCR using pEGFP-ALD-F/R and pcDNA-ALD-F/R (Figure 1A). Sequencing analysis showed that the total length of the open reading frame (ORF) of *NsALD* was successfully acquired, which encoded an alanine dehydrogenase homolog. The results of sequencing analysis revealed that the ORF of the *NsALD* gene (GenBank: 61148960) was 1,188 bp encoding 395 amino acid sequences, with alanine dehydrogenase/PNT, N-terminal domain, and alanine dehydrogenase NAD-binding and catalytic domains, which belong to the NADB-Rossmann superfamily (Figure 1B). For subcellular localization, NsALD was predicted to locate in the mitochondria with 81% expected accuracy using LocTree 3, while it was predicted to locate in the cytoplasm with 94.1% expected accuracy using PSORT II Prediction. No signal peptide or transit peptide was found. Schematic representation of the prosites demonstrated the protein NsALD to comprise two complexity domains (30–163 and 168–377) and nine kinds of functional sites (Figure 1C).

**Figure 2:**
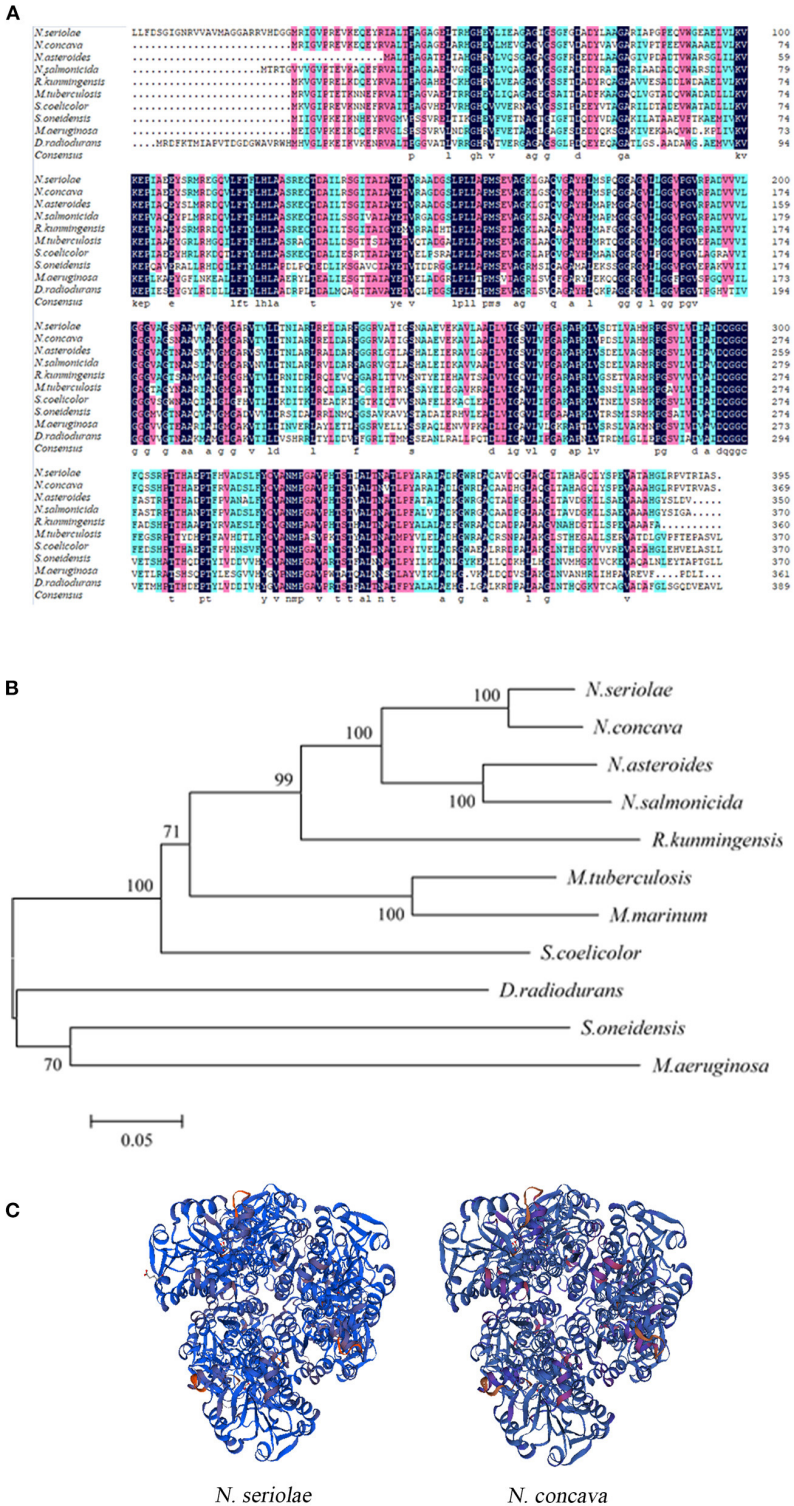
Multiple sequence alignment, construction of a phylogenetic tree, and the three-dimensional structure of *Nocardia seriolae* alanine dehydrogenase (NsALD) protein. **(A)** Multiple alignment of the deduced amino acid sequences of NsALD protein among different species. *Black shaded backgrounds* indicate 100% homology, *pink shaded backgrounds* indicate >75% homology, and *blue shaded backgrounds* indicate >50% homology. **(B)** Construction of a phylogenetic tree among *N. seriolae* and other species with protein NsALD homologous sequences. The protein sequences were aligned using DNAMAN software, and the non-rooted neighbor joining tree was generated by the MEGA 5.0 program. *Number at branch points* indicates bootstrap support. **(C)** Structure analysis of NsALD from *N. seriolae*. The predicted three-dimensional structure of NsALD protein from *N. seriolae* (*left*) and *Nocardia concava* (*right*). The diagrams were generated using SWISS-MODEL online. GenBank accession numbers are shown as follows: *N. seriolae* ZJ0503, WP_033090406.1; *N. concava*, WP_040806553.1; *Nocardia asteroides*, SFM85941.1; *Nocardia salmonicida*, WP_062992005.1; *Rhodococcus kunmingensis*, WP_068269631.1; *Mycobacterium tuberculosis* H37Rv, NP_217296.1; *Mycobacterium marinum*, WP_020728350.1; *Streptomyces coelicolor* A3(2), NP_626044.1; *Shewanella oneidensis* MR-1, YP_007001355.1; *Microcystis aeruginosa*, WP_012268249.1; and *Deinococcus radiodurans* R1, NP_295618.1.

BLAST protein analysis showed that the NsALD amino acid sequence had high homology with other ALD sequences in actinomycetes, with 92.41, 79.05, 76.15, 68.29, 66.67, 62.60, 52.30, 50.68, and 53.66% identity to the ALDs of *Nocardia concava, N. asteroides, N. salmonicida, Rhodococcus kunmingensis, Mycobacterium tuberculosis* H37Rv, *Streptomyces coelicolor* A3(2), *Shewanella oneidensis* MR-1, *Microcystis aeruginosa*, and *Deinococcus radiodurans* R1, respectively (Figure 2A). A biological evolutionary tree was constructed with the amino acid sequences of 12 bacterial ALDs, which shows that NsALD had a rather high homology among the *Nocardia* species (Figure 2B). Structure analysis showed a high similarity between the ALDs from *N. seriolae* and *Nocardia concave* (Figure 2C).

### Detection of Subcellular Localization of NsALD in Transfected Cells

**Figure 3:**
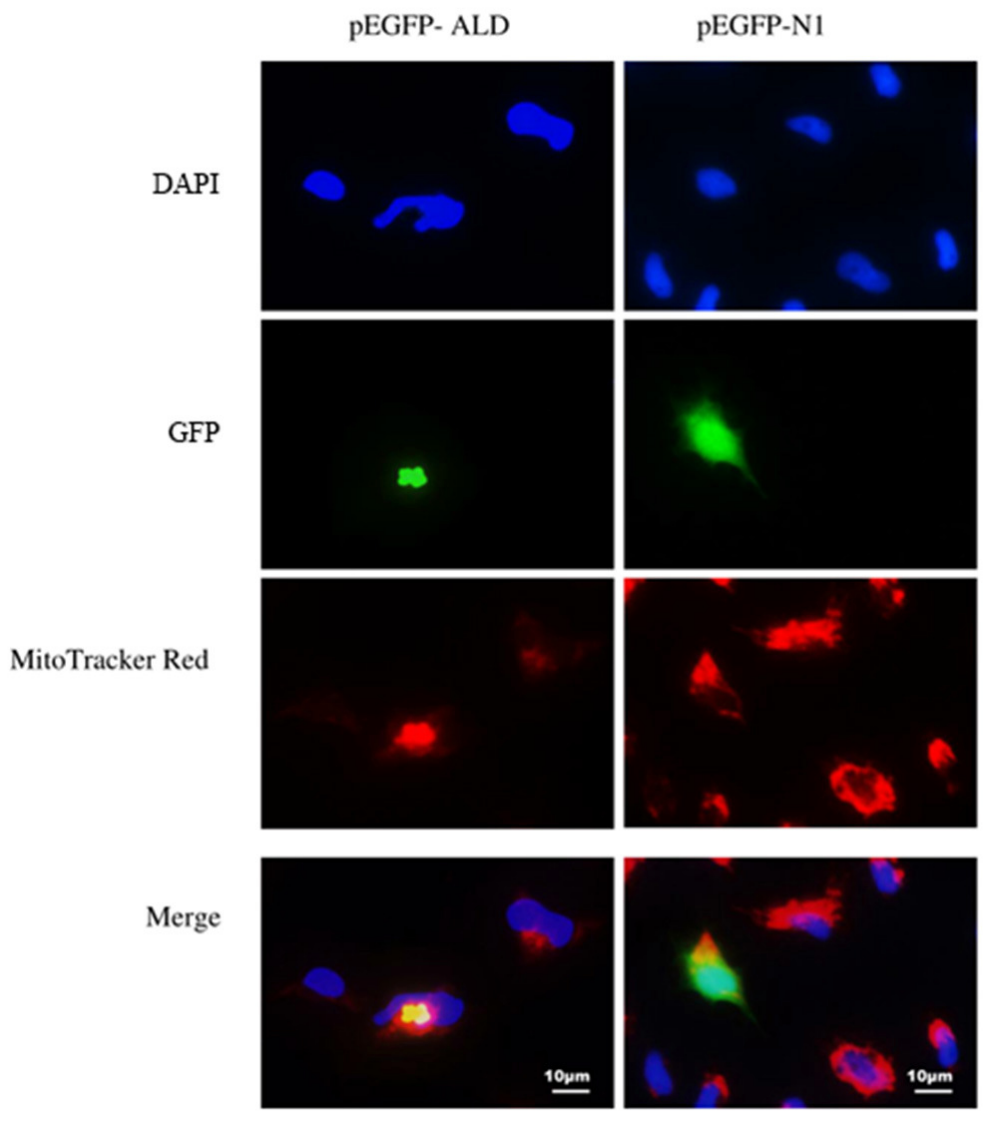
Subcellular localization of the *Nocardia seriolae* alanine dehydrogenase (NsALD) protein in fathead minnow (FHM) cells. Green fluorescence shows ALD-GFP or GFP, red fluorescence shows the mitochondria, and blue fluorescence shows the nucleus. *Left panels* are pEGFP-ALD and *right panels* are pEGFP-N1. In pEGFP-N1-transfected cells, the green fluorescence was dispersed in the whole cell of FHM cells, the mitochondria were distributed in the perinuclear cytoplasm, and the edge of the nucleus was smooth and had no apoptosis characteristics. In pEGFP-ALD-transfected cells, nuclear shrinkage appeared, the mitochondria aggregated near the nucleus, and a strong green fluorescenc of ALD-GFP exhibited an aggregated distribution near the nucleus and overlapped with the distribution of the mitochondria.

To explore the subcellular localization of the NsALD protein, the recombinant plasmid pEGFP-ALD was transfected into FHM cells, which was tested by green fluorescence signals. The nucleus was shown as blue fluorescence and the mitochondria was displayed as red fluorescence. In pEGFP-N1-transfected cells, the green fluorescence was dispersed in the whole cell of FHM cells, the mitochondria were distributed in the perinuclear cytoplasm, and the nucleus margin was smooth and had no apoptosis feature (Figure 3, right). Differently, in pEGFP-ALD-transfected cells, nuclear shrinkage appeared, the mitochondria aggregated near the nucleus, and a strong green fluorescence of ALD-GFP exhibited an aggregated distribution near the nucleus and overlapped with the location of the mitochondria, which indicated that the protein NsALD was co-localized with the mitochondria (Figure 3, left).

### Secreted Protein Identification to NsALD

The secreted proteins of *N. seriolae* were acquired and identified with shotgun mass spectrometry. The results revealed that three characteristic peptide sequences of NsALD (IALTPAGAGELTR, SGITAIAYETVR, and VKEPIAEEYSR) were tested with a confidence ≥99%, which demonstrated that NsALD is a secreted protein of *Nocardia seriolae*.

### Apoptosis Detection Induced by the Overexpression of *NsALD*

**Figure 4:**
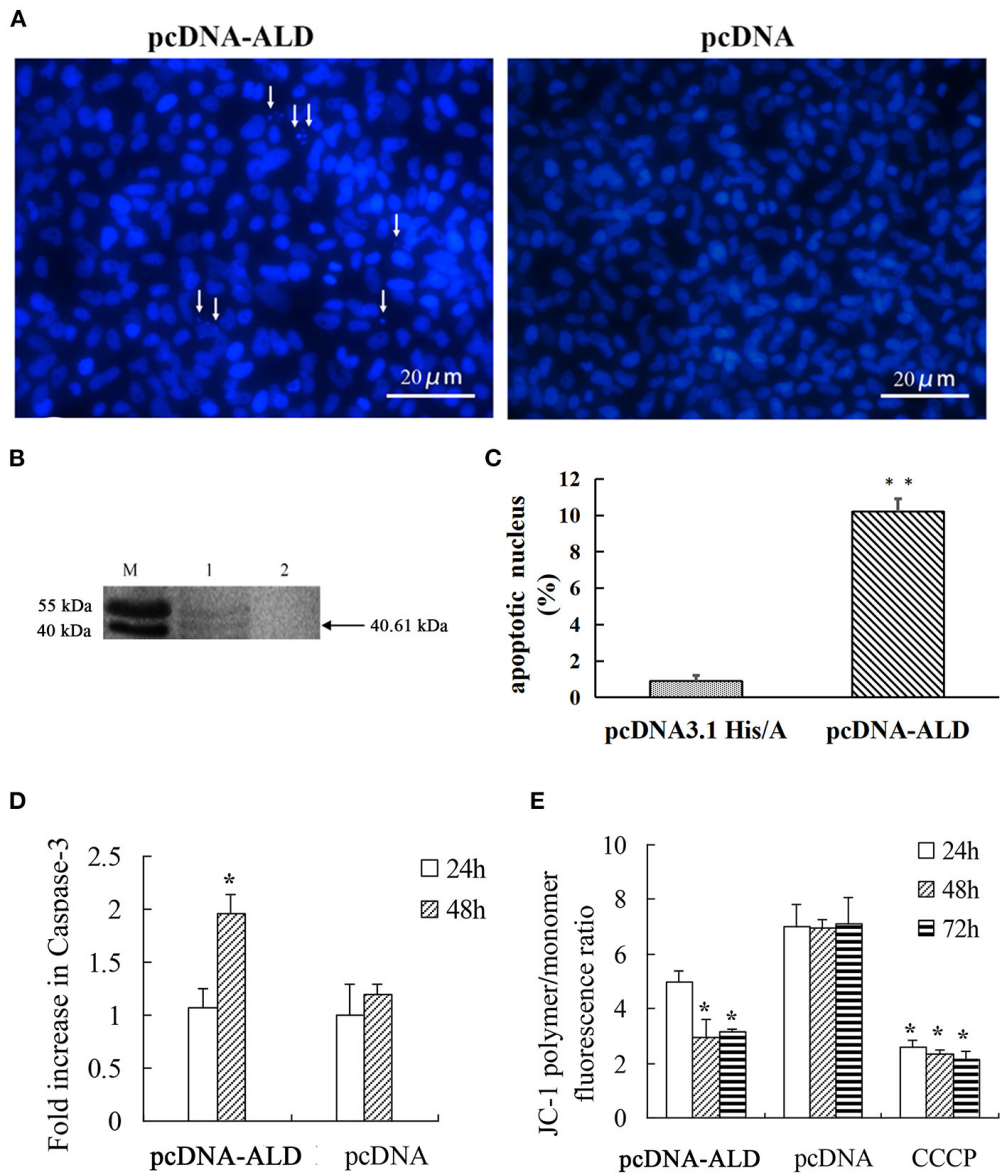
Apoptosis characteristics in pcDNA-ALD-transfected fathead minnow (FHM) cells. **(A)** Overexpression of protein NsALD (*Nocardia seriolae* alanine dehydrogenase) in FHM cells. *White arrows* indicate apoptotic bodies. **(B)** Confirmation of NsALD expression in FHM cells by Western blot. *M*, marker. *1*, NsALD; *2*, pcDNA. **(C)** Percentage of apoptotic body in plasmid pcDNA-ALD- or pcDNA-transfected cells. *Error bars* indicate SD (**p < 0.01). **(D)** Fold increase in caspase-3 after plasmid pcDNA-ALD or pcDNA transfection of FHM cells. FHM cells transfected with plasmids were collected at indicated points after transfection and the levels of cleaved caspase-3 measured. *Error bars* indicate SD (*p < 0.05). **(E)** Detection of ΔΨ_m_ values. FHM cells transfected with plasmid pcDNA-ALD or pcDNA were collected at indicated time points after transfection and the values accessed using JC-1. Untransfected cells treated with carbonyl cyanide *m*-chlorophenylhydrazone (CCCP) were positive controls. Data are expressed as the JC-1 polymer/monomer fluorescence ratio. *Error bars* indicate SD (*p < 0.05).

To explore whether the NsALD protein has an influence on the apoptosis of fish cells, the control plasmid and the recombinant plasmid pcDNA-ALD were transfected into FHM cells. At 48 hpt, several apoptotic bodies can be observed in the transfected FHM cells (Figure 4A), and Western blot showed that the NsALD protein was expressed (Figure 4B). Apoptotic bodies were counted to calculate the apoptosis rate. The result revealed that there were highly significant differences between the experimental group and the control group (Figure 4C). As exhibited in Figure 4D, detection of caspase-3 activity revealed that the maximum value appeared at 48 hpt, which was a 1.63-fold increase compared to that of the control group. Furthermore, the ΔΨ_m_ values of NsALD-overexpressing cells compared with those of control cells were reduced remarkably. The ΔΨ_m_ values of NsALD-overexpressing cells decreased to minimum values at 48 hpt, a decrease of 0.43-fold compared to the control group (Figure 4E).

### Quantitative Detection of Apoptosis-Related Genes in FHM Cells

**Figure 5:**
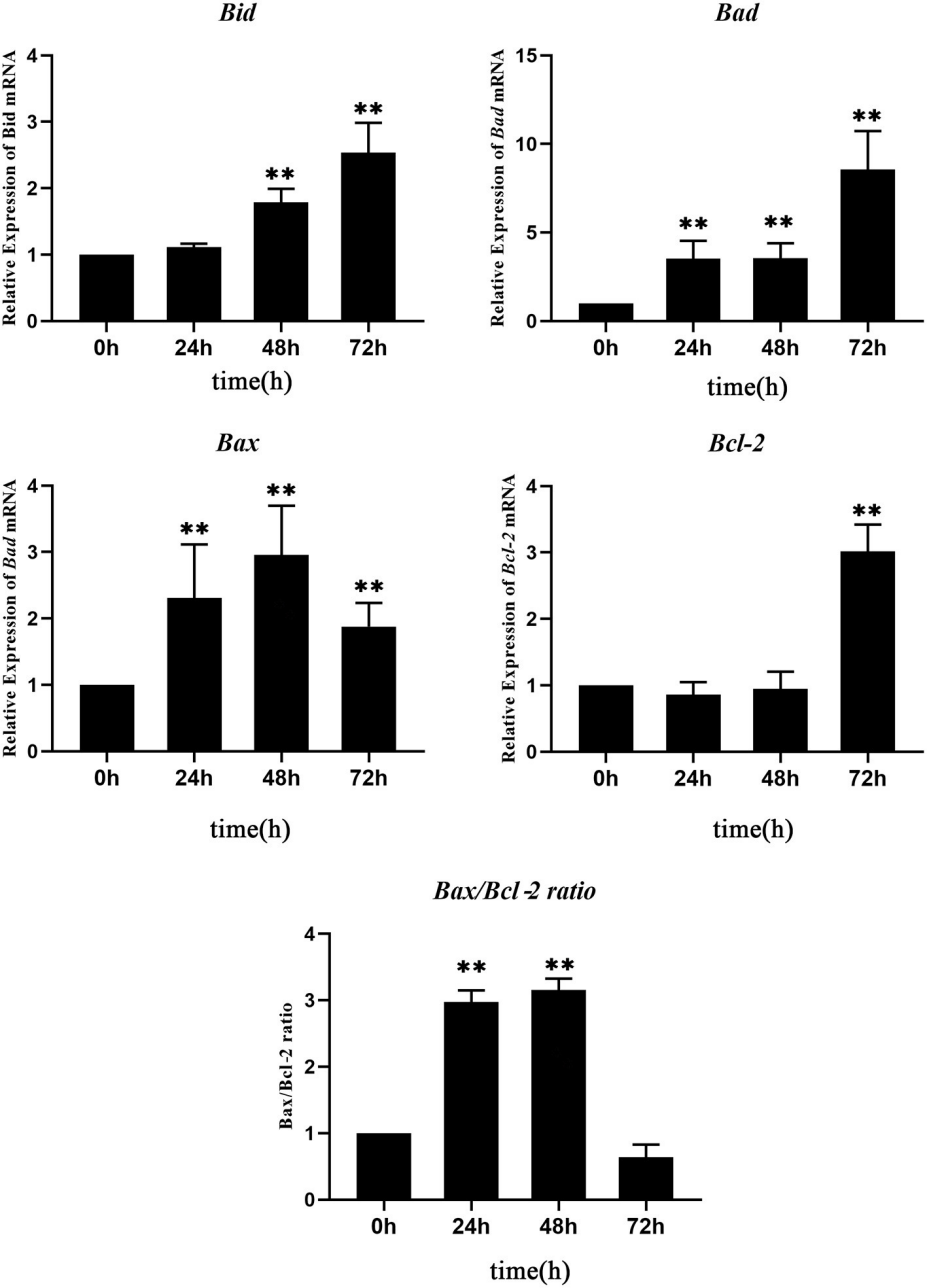
Real-time quantitative PCR (RT-qPCR) analysis of the expressions of apoptosis-related genes in *Nocardia seriolae* alanine dehydrogenase (NsALD)-overexpressing fathead minnow (FHM) cells. Significant differences are indicated by **p < 0.01.

The expression level of each apoptosis-related gene at 0 hpt was considered as the control group. The expressions of *Bad* and *Bax* genes increased significantly at 24–72 hpt, with peak values of 10.5-fold at 72 hpt and 2.9-fold at 48 hpt, respectively. The *Bid* gene increased significantly at 48–72 hpt, reaching a peak value of 2.5-fold at 72 hpt. Interestingly, the expression of *Bcl-2*, which is an anti-apoptotic gene, showed no change during 0–48 hpt, while it increased significantly at 72 hpt. Since a high *Bax*/*Bcl-2* ratio is related to cell apoptosis, the *Bax*/*Bcl-2* ratio was calculated according to their expressions. The *Bax*/*Bcl-2* ratio increased significantly at 24–48 hpt, with a peak value of 10.5-fold at 48 hpt, and then it decreased to 0.5 times that of the control group at 72 hpt (Figure 5). This meant that, although the expression of *Bcl-2* increased significantly, the *Bax*/*Bcl-2* ratio is still low at 72 hpt.

## Discussion

Alanine dehydrogenase (ALD) is a microbial enzyme that catalyzes the reversible oxidation of the deamination of alanine to pyruvate. ALD interconversion between alanine and pyruvate is the core of microbial metabolism (19). ALD was firstly found in *Bacillus subtilis* by Wiame and Pierard (20). The pathogenicity of bacteria is closely associated with its pathogenic factors. However, only a few studies have been conducted on the virulence factor of *N. seriolae*. Interestingly, in some bacteria, ALD was found to be an antigen-secreting protein *in vitro* and was regarded as a virulence factor of pathogenic bacteria. For instance, ALD was found in *M. tuberculosis* culture filtrate and identified as one of its main antigens (21). Transcriptional induction of the *ALD* gene has been observed upon infection of leopard frogs with *Mycobacterium marinum*, which indicated that ALD may be crucial during *M. marinum* infection (22). So far, information about the regulation of ALD and its possible role in pathogenesis is limited (18), and no study related to the ALD of *N. seriolae* (NsALD) has been reported.

In this study, NsALD was obtained and identified as a secreted protein without a signal peptide. It has been reported that the ALD of *M. tuberculosis* is a predominant antigen in its culture filtrate, which means that ALD is a secreted protein of *M. tuberculosis*; the ALD of *M. tuberculosis* also lacks a signal peptide (23). NsALD was found to co-localize with the mitochondria in this research. Some proteins targeting the mitochondria contain a transit peptide, while no transit peptide was found in the NsALD protein by bioinformatic prediction. Mitochondrial targeting of bacterial proteins has been reported. Some proteins from parasitic microorganisms such as rickettsial postulated peptidase (RPP) have been discovered to be similar to the protein subunit of mitochondrial processing peptidase (MPP) (24). Additionally, some bacterial virulence factors contain N-terminal mitochondrial targeting signals (25, 26). The host cells are invaded by certain proteins of Gram-negative bacteria through the type 3 secretion system (TTSS) and are co-positioned with the mitochondria of host cells (25–27). *Helicobacter pylori* VacA-targeted mitochondria was associated with the formation of anion-selective channels in mitochondrial membranes (28). Further research on the mitochondria-targeting mechanism of NsALD is needed.

The mitochondria is an important organelle with various important functions, such as participating in innate and adaptive immunity and acting as the signal center in cell apoptosis (29), and is also the key target of bacterial invasion (30). Most of the identified bacterial proteins targeting the mitochondria are involved in cell apoptosis, which is consistent with the core effect of the mitochondria in apoptosis regulation (31). For example, the *hlyA* gene of enteropathogenic *E. coli* (EPEC) encodes hemolysin, which is targeted to the mitochondria and triggers the apoptosis of host cells *via* mitochondrial pathways (25). In the process of infection with macrophages, the secreted SopA protein of *Salmonella enterica* locates in the mitochondria to activate the caspase-1 independent pathway *via* the TTSS and leads to macrophage death (32). Cytotoxin VacA related to gastric epithelial lesions is a virulence factor of pathogenic *H. pylori* (33). It was found that p34, a subunit of VacA, specifically locates in host mitochondria and induces the release of host cytochrome C, which activates caspase-3 and leads to cell death (34). In this study, typical apoptosis characteristics, such as apoptotic bodies, ΔΨ_m_ values dropping, and caspase-3 activation, were examined in NsALD-expressed cells, which indicated that NsALD induces apoptosis in host cells.

In our previous work, the secreted virulence factors of *N. seriolae* have also been identified to induce cell apoptosis. The phospholipase C of *N. seriolae* was a secreted protein and induced apoptosis in FHM cells (15). The MTSP3141 of *N. seriolae* was proven to be a secreted protein, co-located with the mitochondria, and had a 30-amino acid transit peptide at the N-terminal (35). Glutamyl endopeptidase was a secreted protein without a transit peptide, localized in the mitochondria, and was also a virulence factor of *N. seriolae* that induced apoptosis (36). In addition, the superoxide dismutase (SOD) and histone-like DNA-binding protein (HLP) of *N. seriolae* was also identified as secreted proteins that lead to apoptosis in host cells (7, 13). Taken together, secreted protein-induced apoptosis is associated with *N. seriolae* infection.

According to relevant articles, the main apoptosis pathways in fish are the extrinsic/death receptor pathway (37) and the intrinsic/Bcl-2-regulated/mitochondrial pathway (38). In addition, like mammals, fish have similar functions of conserved members of the Bcl-2 family and inherent apoptosis pathways. Among them, the Bax/Bak-like proteins (Bax, Bak, Bok, Bcl-xs, and Mtd) and the BH3-only proteins (Bad, Bid, Bik, Bim, Bmf, and Noxa) are pro-apoptotic molecules (39). According to the results of the RT-qPCR of the four apoptosis-related genes, three pro-apoptotic genes (*Bax, Bad*, and *Bid*) and the *Bax*/*Bcl-2* ratio were significantly activated at 24–48 hpt, suggesting that NsALD could induce the apoptosis of host cells through the intrinsic/Bcl-2-regulated/mitochondrial pathway.

In this study, NsALD was verified to be a secreted protein of *N. seriolae* by shotgun mass spectrometry and target the mitochondria in fish cells. The overexpression of the NsALD protein caused obvious apoptotic characteristics, such as the increase of the activity of caspase-3, the decrease of the ΔΨ_m_ values in FHM cells, and the significantly induced expressions of pro-apoptotic genes, indicating that NsALD can induce cell apoptosis. This study has laid the foundation for further clarification of the function of NsALD and provided new insights into the understanding of the molecular pathogenicity of *N. seriolae*.

## Data Availability Statement

The original contributions presented in the study are included in the article/Supplementary Material, further inquiries can be directed to the corresponding author/s.

## Ethics Statement

This research did not include live animals. All animal experimental procedures involved were carried out in accordance with the Regulations for Animal Experimentation of GuangDong Ocean University, and the animal facility was based on the National Institutes of Health guide for the care and use of the Laboratory (NIH Publications No. 8023).

## Author Contributions

LX and YLiu designed the experiments. GC, LX, ZT, YLu, and TW performed the experiments. GC, LX, and ZT contributed to analysis. GC and LX wrote the paper. YLiu polished the paper. All authors contributed to the article and approved the submitted version.

## Funding

This work was supported by the National Key Research and Development Project (2020YFD0900201), the Natural Science Foundation of Guangdong Province (2021A1515011222), the Key Research and Development Projects in Guangdong Province (2020B0202010009), and the Shenzhen Dapeng New District special fund for industry development (KJYF202001-08).

## Conflict of Interest

The authors declare that the research was conducted in the absence of any commercial or financial relationships that could be construed as a potential conflict of interest.

## Publisher's Note

All claims expressed in this article are solely those of the authors and do not necessarily represent those of their affiliated organizations, or those of the publisher, the editors and the reviewers. Any product that may be evaluated in this article, or claim that may be made by its manufacturer, is not guaranteed or endorsed by the publisher.
